# Medication Gaps and Antipsychotic Polypharmacy in Previously Hospitalized Schizophrenia Patients: An Electronic Cohort Study in Three Canadian Provinces

**DOI:** 10.3389/fpsyt.2022.917361

**Published:** 2022-06-15

**Authors:** Evyn Peters, Arash Shamloo, Rohit J. Lodhi, Gene Marcoux, Kylie Jackson, Shawn Halayka, Lloyd Balbuena

**Affiliations:** ^1^Department of Psychiatry, University of Saskatchewan, Saskatoon, SK, Canada; ^2^Department of Psychiatry, University of Western Ontario, London, ON, Canada; ^3^Mental Health Outpatient Services, Saskatchewan Health Authority, Prince Albert, SK, Canada; ^4^Independent Researcher, Prince Albert, SK, Canada

**Keywords:** polypharmacy, real world evidence (RWE), treatment guideline implementation, antipsychotics, schizophenia, adherence - compliance - persistance

## Abstract

**Background:**

Real world evidence about antipsychotics focuses on rehospitalization. Modeling the time course of pharmacotherapy would show patients' adherence to medications and physicians' adherence to medication guidelines. We aimed to calculate the cumulative time spent in second generation antipsychotics (SGAs), gaps, antipsychotic polypharmacy, and clozapine in discharged schizophrenia patients.

**Methods:**

Hospitalization and pharmacy dispensing data from 2008–2018 in Manitoba, Saskatchewan, and British Columbia were linked and an electronic cohort (*N* = 2,997) was created (mean follow-up: 49 months, SD = 38). Cohort members were required to have a minimum of 6 weeks medicated with aripiprazole, olanzapine, paliperidone, quetiapine, risperidone, or ziprasidone.

**Results:**

The multistate model predicted that schizophrenia patients accumulated 44 months in SGA monotherapy, 4 months in polypharmacy, 11 months in medication gaps and 17 days in clozapine over a 5-year period. The majority of transitions were between SGA and medication gap. Accumulated time in medication gaps was seven times as much as in clozapine. Each 10% delay in SGA initiation post-discharge was associated with a 2, 1, and 6% higher risk for polypharmacy (95% CI: 1.01–1.02), gap (95% CI: 1.01–1.01), and clozapine (95% CI: 1.04–1.08), respectively.

**Interpretation:**

Schizophrenia patients accumulated more time unmedicated and in polypharmacy compared to clozapine. Either treatment guidelines for schizophrenia are not followed, or real-world challenges hamper their implementation.

## Introduction

Pharmacotherapy remains the mainstay of treatment for schizophrenia (1). Yet with few specific treatment guidelines, prescribers necessarily rely upon clinical acumen in collaboration with patients. High-quality evidence for first-episode psychosis is even scarcer, as ethical considerations discourage placebo-controlled trials (1).

An exception is clozapine for treatment-resistant schizophrenia, which continues to be strongly supported (1, 2). There is also evidence to suggest clozapine for patients with prominent negative symptoms (3, 4), aggression (5), suicidality (6), and comorbid substance use (7). However, clozapine is prescribed less frequently than would be expected by its indications (2, 8, 9), suggesting either an implementation failure or treatment guidelines that overestimate real-world effectiveness. Real world studies on clozapine tend to focus on more severely ill patients, so it is hard to estimate at what point it is introduced in treatment (10).

According to the Maudsley Treatment Review and Assessment Team (TREAT), various practical considerations contribute to the under-prescribing (or delayed initiation) of clozapine (11). Among these are: identifying treatment refractory patients, ascertaining if previous treatment was adequate, establishing the patient's willingness to engage, and weighing the risks vs. benefits of clozapine for each patient (11). These steps require close coordination among different care providers. Patients on their part may refuse blood tests, and this can delay clozapine initiation (12). There are also barriers on the side of clinicians such as fear of serious side-effects, the burden of constant monitoring, and a self-reported lack of competence (12). Among patients who have agreed to clozapine treatment, several reasons are given for discontinuing: intolerable side effects, non-compliance with blood monitoring, and dysfunctional beliefs about clozapine treatment (13).

Advanced statistical techniques such as network meta-analysis have recently been used to rank large numbers of antipsychotics in terms of efficacy and tolerability/safety with data from clinical trials (3, 14). It remains less clear if these rankings inform clinical practice. The extensive selection criteria found in most trials is a constraint to the generalizability of the findings, thereby making it necessary to examine real-world data (15).

Here, we analyzed prescription and hospitalization data for patients with schizophrenia from three Canadian provinces, with the following objectives:

1. Estimate the dispensing frequency of several second-generation antipsychotics (SGA).
2. Estimate the prevalence of (i) initial SGA, (ii) polypharmacy, (iii) unmedicated treatment gaps, (iv) clozapine, (v) rehospitalization, as well as the cumulative time spent and total visits (or returns) to each state.
3. Calculate the probability of transitions between states and how these are affected by socio-demographic characteristics.

## Methods

### Patient Involvement

One-on-one interviews with six persons with schizophrenia and a mental health nurse were held to elicit their lived experience and inform our research questions. After obtaining the results, one patient provided feedback. Recruiting patients, a vulnerable group, was approved by the university ethics board and all participants gave their informed consent and received a small honorarium. Our research was conducted in accordance with the Helsinki declaration of respect for patients.

### Data Sources

This was a retrospective study of administrative data about hospitalizations and pharmacy dispensing data in British Columbia, Manitoba, and Saskatchewan from 2008–2018. Hospitalization data consisted of emergency room visits captured in the National Ambulatory Care Reporting System (NACRS) and inpatient stays in the Discharge Abstract Database (DAD). Psychotropic medications were obtained from the National Prescription Drug Utilization Information System Database (NPDUIS)—a Canada-wide register of publicly funded medications dispensed by pharmacies in the community. Despite this national scope, the provincial (territorial) programs only cover low income people and seniors, except for the provinces in this study. Hospitalization and medication files were provided separately by the Canadian Institute of Health Information (CIHI). We linked these files using a unique person identifier provided by CIHI. Our data captured all mental-health related visits and >85% of all psychotropic medications. The exceptions were medications used in hospital and those covered by federal programs. Also, patients emigrating from the three Canadian provinces may have had their records truncated.

### Cohort Formation

Patients in an electronic register differ importantly from those enrolled in randomized clinical trials. Trial participants satisfy explicit inclusion and exclusion criteria, thereby ensuring that they are a homogenous group. In contrast, patients in our registries only had the common characteristic of visiting the hospital for a mental condition and obtaining medications from a pharmacy. As such, our first task was to identify a more-or-less homogenous cohort in diagnosis and severity of illness. Accordingly, we formed a cohort of patients using their first hospital visit as an index date and applied the following criteria:

1. Schizophrenia diagnosis (ICD-10 codes: F20.1–F20.9).
2. Adult onset (i.e., aged 19+ at index hospital visit, allowing for the possibility that the true illness onset may have been 18 or younger).
3. Medicated for at least 6 weeks with one of the following SGAs after the hospital visit: aripiprazole, olanzapine, paliperidone, quetiapine, risperidone, and ziprasidone.

We further applied the following criteria in order to capture patients with less serious presentations (i.e., excluding resistant or seriously ill patients):

4. First hospital visit did not contain an ICD code for intentional self-injury or poisoning (X60–X84).
5. Patient was not already on SGA polypharmacy (i.e., not medication resistant).
6. Patient was not already on clozapine or a less-commonly dispensed SGA.

**Figure 1:**
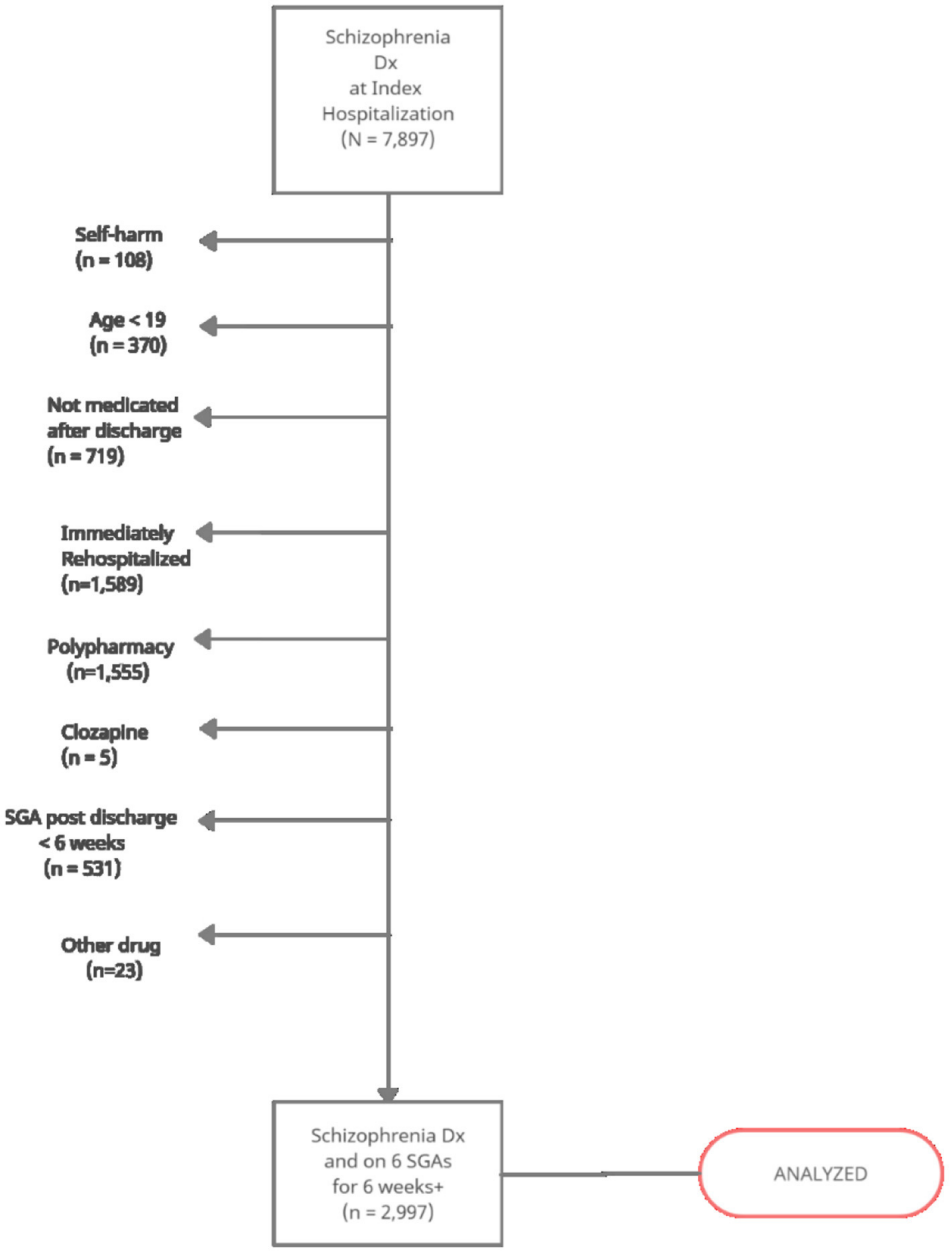
Strobe diagram of a cohort of schizophrenia patients and selection criteria.

Applying these criteria to the 7,897 patients with a schizophrenia diagnosis at the index visit yielded a cohort of 2,997 people (Figure 1). The socio-demographic characteristics of these people at their index visit are summarized in Table 1. For each cohort member, SGA refills (and rehospitalization, if applicable) were followed until February 2019 or loss to follow-up, whichever came first. Cohort members had a mean follow-up time of 49 months (SD = 38) and this did not differ by province.

**Table 1:** Demographic profile of schizophrenia patients at index hospitalization in three Canadian provinces.

**Variable**	**Manitoba**	**Saskatchewan**	**British Columbia**
*N*	609 (20)	272 (9)	2,116 (71)
Mean age (SD)	48 (19)	51 (19)	44 (17)
Sex			
Male (%)	367 (60)	156 (57)	1,284 (61)
Female (%)	242 (40)	116 (43)	832 (39)
Initial SGA			
Aripiprazole (%)	22 (4)	11 (4)	167 (8)
Olanzapine (%)	230 (38)	59 (22)	558 (26)
Paliperidone (%)	15 (2)	11 (4)	218 (10)
Quetiapine (%)	115 (19)	73 (27)	336 (16)
Risperidone (%)	220 (36)	107 (39)	800 (38)
Ziprasidone (%)	7(1)	11 (4)	37 (2)
Residence			
Rural, remote, or unclassified area of residence	90 (15)	46 (17)	177 (8)
Urban	519 (85)	226 (83)	1,939 (92)
Mean days to first SGA dispense after discharge from index hospitalization (SD)	45 (144)	54 (209)	37 (131)
Mean months to first clozapine dispense (SD)^*^	30 (34)	13 (14)	41 (28)

* *Calculated only for 57 people who were ever put on clozapine; Number (% of total patients): Manitoba: 9 (1.5), Saskatchewan: 3 (1.1), B.C: 45 (2.1)*.

### Classification of Treatment States

Each person in the cohort had a longitudinal record of hospitalizations and medication refills. We sorted these records from earliest to most recent. We were interested in four transient states: (1) initial antipsychotic, (2) polypharmacy, (3) unmedicated (treatment gaps), and (4) clozapine. Each person had to have spent at least 6 weeks on SGA monotherapy. This was based on the guideline that response to treatment (if it happens at all) can typically be observed within the first 6–8 weeks (16). The terminal state was rehospitalization. From SGA monotherapy, each person could progress to any of the transient States (2–4), with repeated visits. Follow-up ended when a person was rehospitalized, the person ran out of data, or the cut-off of CIHI data was reached. In the latter two cases, the person was considered lost to follow-up. The assignment of states, especially gaps and polypharmacy (discussed below), was greatly facilitated by the Stata newspell package (17).

### Definition of Polypharmacy, Clozapine, and Rehospitalization States

A patient was assigned to the polypharmacy state if two or more psychotropic medications overlapped during follow-up. These psychotropic medications were not limited to the six SGAs in State 1 but included asenapine, chlorpromazine, flupentixol, fluphenazine, haloperidol, levomepromazine, lithium, loxapine, lurasidone, perphenazine, pimozide, pipotiazine, prochlorperazine, sulpiride, trifluoroperazine, and zuclopenthixol. If clozapine overlapped with another medication, this period was assigned to clozapine. Rehospitalization in this study was defined as a hospital visit associated with a mental health condition (ICD F code) or self-harm (ICD X60–X84).

### Treatment Gap Calculation

Since all hospitalizations and all psychotropic refills were captured, the follow-up period for each person could be classified into the five states above, subject to the limitation that we did not have quantity and dose at each refill. For this reason, we could not be sure that a person possessed adequate medication during the period between refills, or if there was a gap for which a person was unmedicated. People filled prescriptions at irregular intervals. According to a paper about estimating medication adherence, the grace period between consecutive refills, in various studies, ranges from 15 to 120 days (18). Based on typical prescribing practices, we fixed the grace period at 30 days. Treatment gap was therefore operationally defined as the period starting from day 31 to the date of the subsequent refill (Supplementary Figure S1).

### Analysis

We fitted a multistate survival model (MSM) using the R package msm (19). This was based on several considerations: (1) The msm package is capable of handling continuous (vs. discrete) time transitions. (2) It handles intermittently observed events (i.e., refills). (3) The msm package allows for cyclic transitions. A MSM is comparable to a simple survival model except that the hazard is calculated for transitions between transient states (i.e., SGA, polypharmacy, gap, and clozapine) and from transient states to the absorbing state (rehospitalization). For a schematic of the states and transitions in our model, please refer to Supplementary Figure S2. We had four candidate predictor variables: age at index hospitalization, the interval between hospital discharge and the first pharmacy dispense date, rural/remote/unclassified area of residence vs. urban, and gender.

## Results

### State Prevalence Up to 60 Months

As Table 2 shows, the proportion of schizophrenia patients who were on an SGA was close to 70% at up to 5 years. However, at any single time except at baseline, 16–20% of patients had a gap in treatment and 5–7% were on polypharmacy. The total proportion of patients who received clozapine (whether by itself or with another antipsychotic) never reached 3% over the entire follow-up (*n* = 57 people). Close to 5% of remaining cohort members were rehospitalized at 5 years.

**Table 2:** Number (%) of patients in a stage by follow-up time (months). The denominator is the total number of patients remaining in the cohort at a given month.

**Follow-up time** **in months**	**SGA**	**Polypharmacy**	**Medication Gap**	**Clozapine**	**Rehospitalized**
0	2,997 (100.0)	0 (0.0)	0 (0.0)	0 (0.0)	0 (0.0)
6	2,067 (79.5)	96 (3.7)	430 (16.5)	4 (0.2)	4 (0.2)
12	1,757 (75.8)	95 (4.1)	446 (19.2)	7 (0.3)	12 (0.5)
18	1,543 (73.0)	116 (5.5)	423 (20.0)	16 (0.8)	17 (0.8)
24	1,394 (72.3)	108 (5.6)	388 (20.1)	18 (0.9)	21 (1.1)
30	1,240 (71.3)	108 (6.2)	347 (19.9)	19 (1.1)	26 (1.5)
36	1,113 (70.3)	120 (7.6)	303 (19.1)	16 (1.0)	32 (2.0)
42	995 (69.5)	100 (7.0)	283 (19.8)	19 (1.3)	35 (2.4)
48	874 (67.6)	105 (8.1)	256 (19.8)	19 (1.5)	39 (3.0)
54	817 (69.1)	83 (7.0)	218 (18.4)	21 (1.8)	44 (3.7)
60	740 (69.2)	73 (6.8)	184 (17.2)	23 (2.2)	49 (4.6)

Over a 5-year (60-month) period, the accumulated time spent in each of the states were: 44 months in initial SGA, 4 months in polypharmacy, 11 months in medication gaps, and 17 days in clozapine. On average, patients had 10 spells of SGA monotherapy, 1 spell of polypharmacy, 10 untreated spells and <1 clozapine spell. SGA monotherapy and gaps tend to cycle back-and-forth as shown in Supplementary Figure S3.

### Transition Probabilities Between States

At 3 months, there was an 18% percent chance of a gap in treatment given that one was on an SGA. Given that a patient has a medication gap, returning to an SGA was most likely but there was a 22% chance of continuing to be unmedicated. People on clozapine had a 73% chance of remaining on clozapine. The probability of rehospitalization at 3 months was close to zero (Table 3).

**Table 3:** Probability of transitioning to (remaining in) a state from a given state at 3 months.

**From / To**	**SGA**	**Polypharmacy**	**Gap**	**Clozapine**	**Rehospitalized**
SGA	78	4	18	0	0
Polypharmacy	41	46	12	0	0
Gap	72	5	22	0	0
Clozapine	16	1	10	73	0
Rehospitalized	0	0	0	0	1

*Cell entries are percentages*.

The transition probabilities including loss to follow-up for the entire follow-up period is depicted in Supplement Figure S3. Over the entire follow-up period, only 57 patients (1.9%) ever received clozapine and 64 people (2.1%) were rehospitalized.

### Effect of Socio-Demographic Covariates on Transitions From SGA

Here we focus on how socio-demographic characteristics modify the transitions from SGA, since this is the initial state. See Supplementary Table S1 for the complete results. Each 10% increase after age 19 represented a 3, 2, and 7% risk reduction for polypharmacy, gap, and clozapine, respectively. Each 10% delay in first SGA dispense after discharge was associated with a 2, 1, and 6% higher risk for polypharmacy, gap, and clozapine, respectively. Living in a non-urban area was associated with a 33% lower risk of polypharmacy, a 22% higher risk of a medication gap, and 27% lower risk of being treated with clozapine. Female patients, compared to males had a 20% higher risk of transitioning to polypharmacy from an initial SGA. Female patients on polypharmacy were 88% more likely to have a medication gap. None of the variables predicted rehospitalization from any state.

## Discussion

We had two main findings: (i) schizophrenia patients accumulated substantial periods without medication (treatment gaps), and (ii) patients are on polypharmacy 7 times as long as they are on clozapine.

Although we did not have access to the reasons for treatment gaps, we offer two possibilities. First, patients may have recovered from their illness so as not to require medication, but relapsed sometime later. Up to 20% of schizophrenia patients only experience a single episode as noted in the Canadian Schizophrenia Guidelines (1). This is also consistent with unmedicated rates in naturalistic longitudinal studies in the US (20) and Finland (21). In these studies, ongoing medication was associated with disease severity, and most of the unmedicated patients were clinically stable (20, 21). Therefore, some treatment gaps may reflect periods of recovery and prudent prescribing on the part of physicians.

Alternatively, patients may have needed medications but stopped taking them because of tolerability, logistical, or financial reasons. This fits with the 22% higher risk of treatment gaps among non-urban dwellers from an initial SGA and a 77% higher risk of a gap from polypharmacy (Table 4). Adherence to medications is influenced by patient insight into their illness, perceived efficacy of the medication, family support, and the availability of case managers (22, 23). Patients in non-urban areas may have more limited contact with physicians, community health managers, and health services in general.

**Table 4:** Hazard ratios (95% CI) on selected transitions for four covariates.

**From:**	**To:**	**Index Age (each 10 pct increase from 19 years)**	**Initial Medication Delay (each 10 percent increase from discharge)**	**Rural/Remote/** **Unclassified vs. Urban (reference)**	**Female vs.** **Male (reference)**
SGA	Polypharmacy	0.97 (0.96–0.97)^*^	1.02 (1.01–1.02)^*^	0.67 (0.55–0.81)^*^	1.20 (1.08–1.32)^*^
SGA	Gap	0.98 (0.98–0.98)^*^	1.01 (1.01–1.01)^*^	1.22 (1.16–1.27)^*^	0.99 (0.96–1.02) n.s.
SGA	Clozapine	0.93 (0.91–0.95)^*^	1.06 (1.04–1.08)^*^	0.73 (0.26–2.06) n.s	0.72 (0.36–1.44) n.s.
Polypharmacy	Gap	0.98 (0.97–0.98)^*^	1.00 (1.00–1.00) n.s.	1.77 (1.42–2.20)^*^	1.88 (1.63–2.17)^*^
Polypharmacy	Clozapine	0.93 (0.89–0.97)^*^	1.03 (1.00–1.07)^*^	0.93 (0.14–6.11) n.s.	1.21 (0.40–3.63) n.s.
Gap	Clozapine	0.97 (0.96–0.98)^*^	1.04 (1.04–1.05)^*^	0.40 (0.19–0.85)^*^	Not entered

The two left columns represent the outcome, third to sixth columns are predictors that modify the baseline hazard. Predictor variables are entered simultaneously. Asterisk (*) indicates significance at p = 0.05.

Regarding affordability, it is estimated that 10% of Canadians cannot afford out-of-pocket medication expenses (24). Up to 80% of people with schizophrenia are unemployed (25), so it is possible that some medication gaps are a result of poverty. Unlike universal healthcare coverage, prescription medications are left for provincial governments to decide (26). According to the schizophrenia patients we interviewed, affordability was not a problem because clozapine was paid for by the Saskatchewan provincial government. However, they were generally unable to join the job market—even if they wanted—because their income would count against what they receive from the government.

Although the prevalence of polypharmacy was lower than other studies (27, 28), patients in this cohort were still 7 times as likely to be on polypharmacy as on clozapine. This likely reflects the extent that clozapine is underutilized. Notably, a Canadian study noted that polypharmacy is more prevalent than any antipsychotic monotherapy (29). In the US, <5% of schizophrenia patients were treated with clozapine in 2008 (30)—still higher than the 2% of patients in this cohort. By comparison, up to 50% of schizophrenia patients are on polypharmacy (31) despite the lack of compelling evidence for its efficacy (32). A high polypharmacy rate therefore may therefore represent a gap between treatment guidelines and implementation.

The single biggest barrier to clozapine utilization is probably administrative burden associated with patient monitoring (33). A mental health nurse we interviewed stated that a brand name manufacturer of clozapine relieves some of the administrative burdens by monitoring the patients' blood test results for signs of neutropenia. Patient refusal to initiate (or continue) clozapine treatment may have also contributed to underutilization (12, 34). The three provinces varied significantly with regard to clozapine initiation, with British Columbia waiting more than 3 years on average. By comparison, a study of outpatient schizophrenia patients in Manitoba reported that clozapine was initiated at 8.9 years for males and 7.7 years for females, on average (35). Two-thirds of them had been on 3 or more antipsychotics before the switch to clozapine (35). Delayed initiation of clozapine treatment—perhaps by lingering in mono- or polypharmacy—tends to reduce its efficacy (36).

With evidence-based guidelines being recommendatory, they cannot compel physicians and patients to use clozapine. However, a softer approach that eases administrative burden, provides logistical support, and alleviates patient and clinician concerns has been recommended (37). There is a debate about the ethics of offering monetary rewards for adherence to medications, and a few randomized control studies have been undertaken (38, 39). But the possibility that such an incentive is tantamount to coercion has made it a contentious topic (40).

The finding that older people were at lower risk of polypharmacy, unmedicated periods, and clozapine could mean that they had later onset of the disease. Younger age of onset tends to be associated with a worse prognosis (41). The finding that delay to the first pharmacy claim from hospital discharge increases the risk for polypharmacy, unmedicated periods, and clozapine treatment has implications for the provision of health services. Transitioning from hospital to the community is known to be a vulnerable period in which medication compliance is paramount (42, 43). The use of depot antipsychotics and intensive community treatment programs may improve adherence and prognosis (44–46).

Female patients had higher risk of polypharmacy. Similar results have been reported elsewhere (28, 47, 48) but not invariably (49, 50). Schizophrenia has earlier onset in men than in women and earlier onset is associated with a more severe course of illness (5). Some have reported that women are more adherent to treatment than men (5–7), so we are unsure if this finding reflects a gender difference per se or confounding by other variables.

Our findings are subject to several limitations. We did not know the true age of schizophrenia onset and used age at index hospitalization as a proxy. Given the mean age at index hospitalization (>40 years), it is highly improbable that patients in the cohort were first-onset cases. Since they probably lived with schizophrenia for a decade or more, it is more surprising that clozapine was dispensed to so few and so late in the treatment. Unfortunately, the data cannot distinguish if this is a result of under-prescribing or patients refusing to accept clozapine medication—something that needs to be further studied. Our data sources did not contain clinical notes and symptoms, so we did not have indications for the antipsychotic prescriptions, and the reasons for polypharmacy. Likewise, we could not examine the effect of gaps and polypharmacy on symptoms. Some periods classified as polypharmacy may have been gradual transitions from one SGA to another. Earlier Canadian studies have estimated the prevalence of polypharmacy upon hospital discharge at 19 to 40 percent (29, 51, 52). These are consistent with our finding that polypharmacy is more prevalent than treatment with clozapine. Finally, our data did not include mortality—this may have contributed to the low re-hospitalization rate.

## Data Availability Statement

The data used in this paper were acquired from the Canadian Institute of Health Information, with the agreement of the provinces of British Columbia, Saskatchewan, and Manitoba. The terms of the agreement prohibit the sharing of data without the prior consent of the provinces. Requests to access these datasets should be directed to Smriti Fernandez, smfernandez@cihi.ca, www.cihi.ca.

## Author Contributions

LB, GM, and RJL contributed to the design of the study. LB and AS performed the statistical analysis. EP wrote the introduction and discussion sections. EP and LB presented preliminary results at a meeting and gathered comments. LB wrote the methods and results sections. SH, EP, RL, KJ, and GM contributed to the interpretation of results. All authors contributed to the submitted version. LB, GM, KJ, and RJL acquired funding and the data. All authors contributed to the article and approved the submitted version.

## Funding

This work received funding from the Saskatchewan Center for Patient Oriented Research through a postdoctoral fellowship to Dr. Arash Shamloo.

## Conflict of Interest

LB received a research grant from AA Pharma. AA Pharma did not have a role in the design, data collection, analysis, interpretation, and writing of the paper. The remaining authors declare that the research was conducted in the absence of any commercial or financial relationships that could be construed as a potential conflict of interest.

## Publisher's Note

All claims expressed in this article are solely those of the authors and do not necessarily represent those of their affiliated organizations, or those of the publisher, the editors and the reviewers. Any product that may be evaluated in this article, or claim that may be made by its manufacturer, is not guaranteed or endorsed by the publisher.
