# Supplementary material for: Medication Gaps and Antipsychotic Polypharmacy in Previously Hospitalized Schizophrenia Patients: An Electronic Cohort Study in Three Canadian Provinces
Source: Front Psychiatry. 2022 Jun 15;13:917361. doi: 10.3389/fpsyt.2022.917361 (PMC9243750; doi:10.3389/fpsyt.2022.917361)
Supplement: Supplementary file 1 [file Presentation_1.pdf]

Online Supplement to "*Medication gaps and antipsychotic polypharmacy in previously hospitalized schizophrenia patients: an electronic cohort study in three Canadian provinces*"

Evyn Peters, Arash Shamloo, Rohit J. Lodhi, Gene Marcoux, Kylie Jackson, Shawn Halayka, Lloyd Balbuena

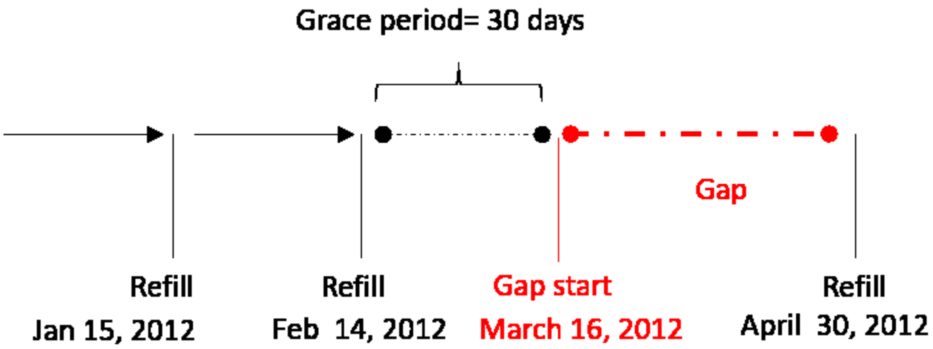

Figure S1: Patient X had three observed psychotropic drug refills. There was a long interval between the refill on February 14th and the subsequent one. We handled such instances by fixing the grace period to 30 days. Unlike the three other refills, March 16th is an imputed date that is based on Sikka et al. (15) and a consensus by the authors.

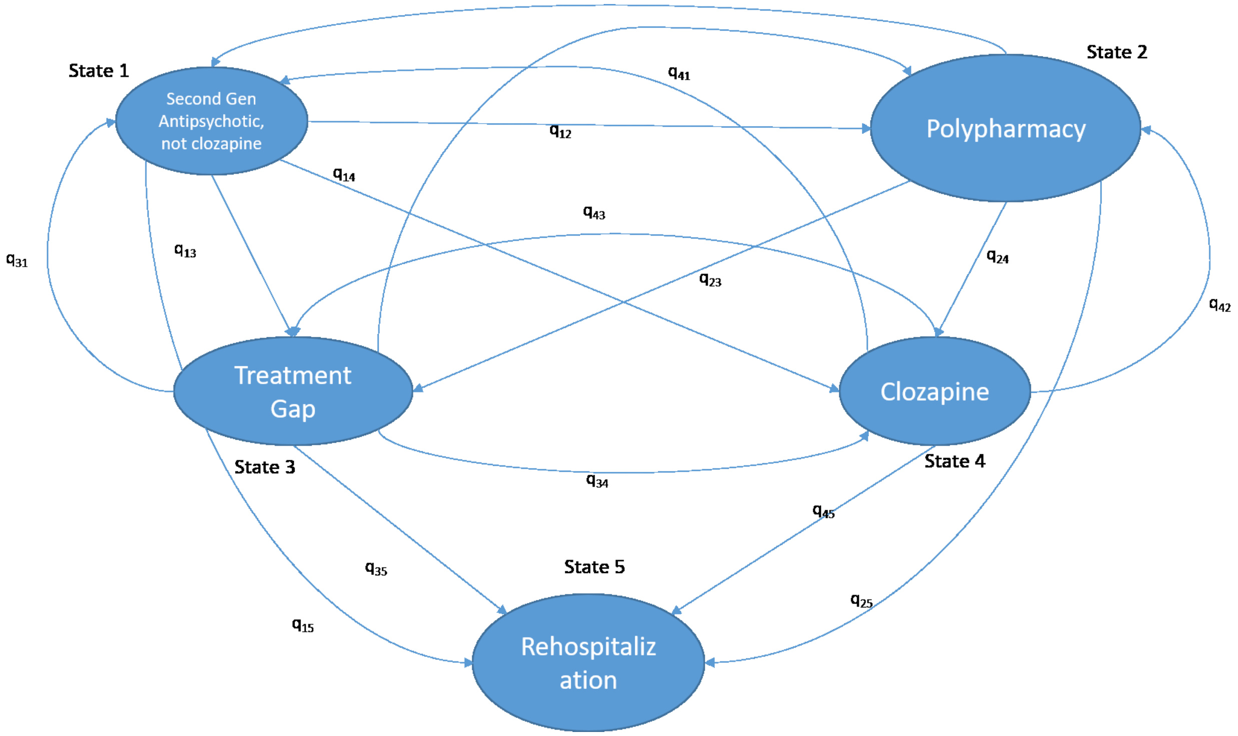

Figure S2: Medication states and transitions. The five medication states are represented by ovals. The arrows labeled q_{rs} indicate a transition from state r to state s. These q's are collected in a matrix Q which is the basis for calculating the likelihood of transitions, prevalences over time, and hazard ratios.

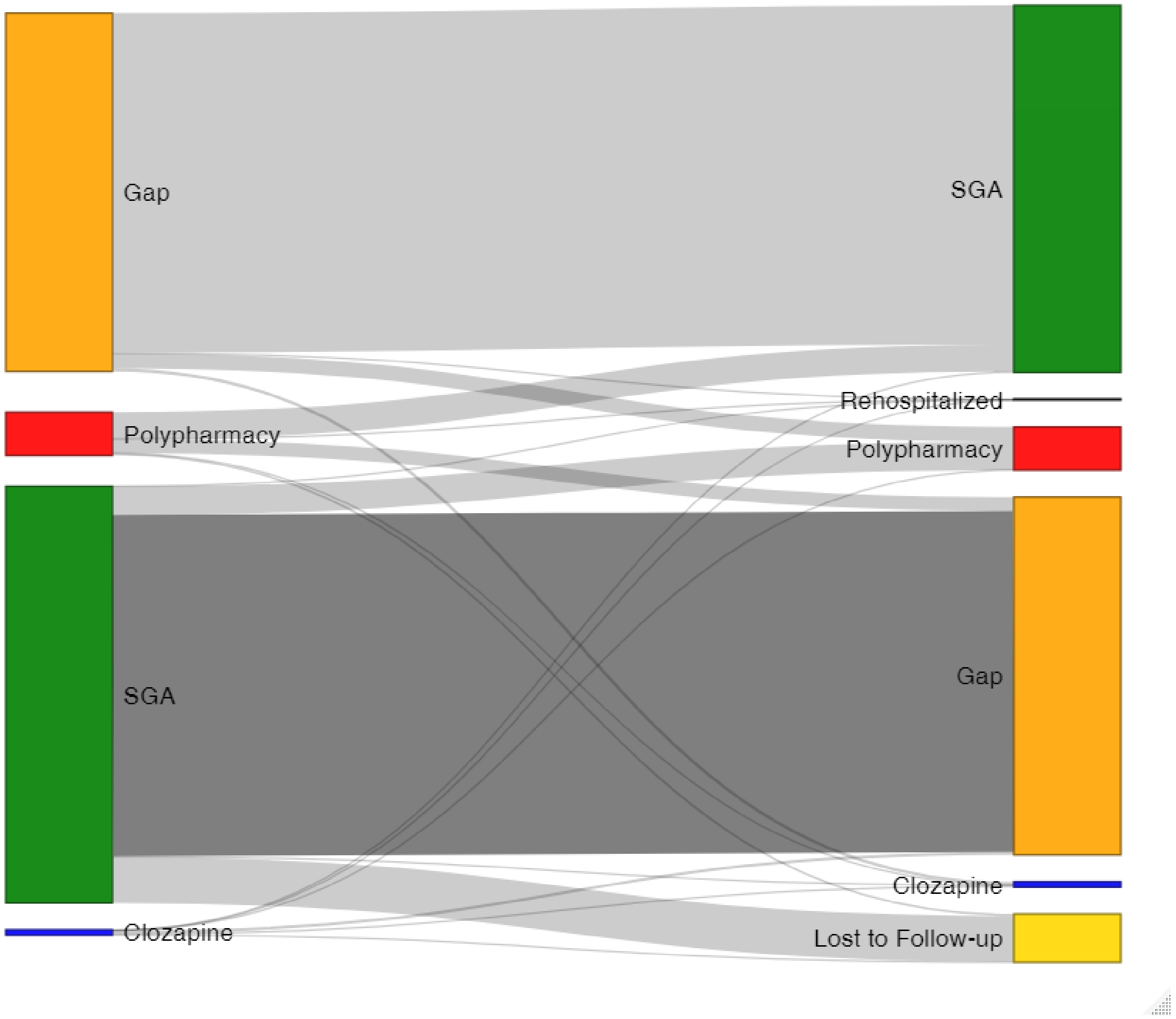

Figure S3: Sankey Diagram of State Transitions. *Origin states are on the left and destination states are on the right. The bar heights for each state are in proportion to visits. The width of gray threads in the middle indicates the flow volume from an origin state to a destination state.*

Table S1: Hazard ratios (95% CI) on all transitions for four covariates (where entered)

From:	To:	Index Age (each 10 pct increase from 19 years)	Initial Medication Delay (each 10 percent increase from discharge)	Rural/Remote/ Unclassified vs Urban (reference)	Female vs Male (reference)
SGA	SGA	Reference	Reference	Reference	Reference
SGA	Poly-pharmacy	0.97 (0.96-0.97)*	1.02 (1.01-1.02) *	0.67 (.55-.81) *	1.20 (1.08-1.32) *
SGA	Gap	0.98 (0.98-0.98) *	1.01 (1.01-1.01) *	1.22 (1.16-1.27) *	0.99 (0.96-1.02) n.s.
SGA	Clozapine	0.93 (0.91-0.95) *	1.06 (1.04-1.08)*	0.73 (0.26-2.06) n.s	0.72 (0.36-1.44) n.s.
SGA	Rehospital-ization	0.99 (0.89-1.11)	1.04 (0.98-1.10)	0.98 (0.03-29.78)	0.98 (0.12-7.80)
Poly-pharmacy	Poly-pharmacy	Reference	Reference	Reference	Reference
Poly-pharmacy	SGA	0.98 (0.98-0.99)*	1.00 (1.00-1.00)	0.73 (0.59-0.90)*	1.13 (1.02-1.26)*
Poly-pharmacy	Gap	0.98 (0.97-0.98)*	1.00 (1.00-1.00)	1.77 (1.42-2.20)*	1.88 (1.63-2.17)*
Poly-Pharmacy	Clozapine	0.93 (0.89-0.97)*	1.03 (1.00-1.07)*	0.93 (0.14-6.11)	1.21 (0.40-3.63)
Poly-Pharmacy	Rehospital-ization	1.04 (0.97-1.11)	1.01 (0.98-1.04)	1.08 (0.17-6.92)	0.91 (0.32-2.57)
Gap	Gap	Reference	Reference	Reference	Reference
Gap	SGA	1.02 (1.02-1.02)*	1.00 (1.00-1.00)	1.28 (1.22-1.33)*	Not entered
Gap	Poly-Pharmacy	1.00 (0.99-1.01)	1.00 (1.00-1.00)	1.17 (0.93-1.45)	Not entered
Gap	Clozapine	0.97 (0.96-0.98)*	1.04 (1.04-1.05)*	0.40 (0.19-0.85)*	Not entered
Gap	Rehospital-ization	Not entered	Not entered	Not entered	Not entered

Note: The two left columns represent the outcome, third to sixth columns are predictors that modify the baseline hazard. Predictor variables are entered simultaneously. Asterisk (*) indicates significance at p = .05
