# Supplementary material for: Medication Gaps and Antipsychotic Polypharmacy in Previously Hospitalized Schizophrenia Patients: An Electronic Cohort Study in Three Canadian Provinces
Source: Front Psychiatry. 2022 Jun 15;13:917361. doi: 10.3389/fpsyt.2022.917361 (PMC9243750; doi:10.3389/fpsyt.2022.917361)
Supplement: Supplementary file 2 [file Presentation_2.pdf]

***Online Supplement to: Medication gaps and antipsychotic polypharmacy in previously hospitalized schizophrenia patients: an electronic cohort study in three Canadian provinces*"**

Evyn Peters, Arash Shamloo, Rohit J. Lodhi, Gene Marcoux, Kylie Jackson, Shawn Halayka, Lloyd Balbuena

Guidance for Reporting Involvement of Patients and Public (GRIPP) 2: Short-form checklist

Section and Topic	Item	Reported
Aim	The aims were informed by prior discussions with patients before we obtained the data	Page 4
Methods	We interviewed 6 persons diagnosed with schizophrenia. We first elicited their lived experiences. We subsequently asked about their experience of medications and their inputs helped form our research questions. The patients were compensated for their expertise in the lived experience of schizophrenia.	Page 4
Study Results	After the analysis, one patient was further invited to provide additional perspective about our results.	Page 12
Discussion and conclusions	Patient inputs contributed to an understanding of real world challenges	Page 13
Reflections / critical perspective	The patients interviewed were all on clozapine. It would have been helpful if we had also interviewed those who were on polypharmacy.<br><br>Having the support of community health nurses greatly facilitated the recruitment of patients.	N/A
